# A Resistance Framework for Racially Minoritized Youth Behaviors During the Transition to Adulthood

**DOI:** 10.1111/jora.12792

**Published:** 2022-08-18

**Authors:** Dawn T. Bounds, Patricia D. Posey

**Affiliations:** ^1^ University of California Irvine; ^2^ University of Chicago

**Keywords:** resistance, transition to adulthood, marginalization, racially minoritized youth

## Abstract

The transition from adolescence to adulthood is a challenging time marked by rapid changes in relational connections, housing status, and academic or work trajectories. We emphasize how structural inequality shapes racially minoritized youth behaviors and center the potential for resistance, arguing that a resistance lens allows us to deepen our understanding of the transition to adulthood for racially minoritized youth. Throughout the paper, we include research on how racially minoritized youth experience marginalizing institutional structures concurrently across multiple systems and their resulting behaviors. We end with the clinical and research implications of a resistance framework to illuminate resistance‐informed responses such as rethinking risk and creating spaces for youth‐led self‐making, youth–adult partnerships to scaffold transitions, and cultivating youth activism.

Racially minoritized youth have shown increasing concern and commitment to standing up to injustices, advocating for policy change, and organizing community members. Notably, George Floyd's death amidst the COVID‐19 pandemic highlighted the pervasive impact of racism on the well‐being of racially minoritized communities (Addo, 2020). For many racially minoritized youth (e.g., Black, Indigenous, and people of color) who are systematically marginalized in the United States (US) due to their race/ethnicity, the protests and rallies about Mr. Floyd's death were their first‐time attending political demonstrations (Retta, 2021). Even though racially minoritized youth under 18 make up over half of the youth in the United States (Frey, 2021), their ability to engage in acts and varying types of resistance is shaped by racial marginalization rooted in U.S. institutions that uphold systems of oppression that create, sustain, and exacerbate negative life conditions, and undermine the well‐being of racially minoritized youth. By resistance, we mean youth behaviors that defy the traditional roles, norms, or responses encouraged by these institutions. For racially minoritized youth transitioning into adulthood (ages 16–24), the multidimensional power imbalance systematically experienced across institutions is racial, economic, and political (Causadias & Umaña‐Taylor, 2018) which means resistance can be protest or voting or more subtle such as failure to adhere to specified rules at work (e.g., tardiness, working slow, or threatening to quit) or the creation of an online subculture (Rosales & Langhout, 2020). Racial marginalization compounds the power imbalances that affect racially minoritized youth's ability to meet the developmental and self‐actualization tasks prescribed to produce a healthy, thriving, productive citizen.

An ecological systems model (Bronfenbrenner, 1986, 1992) can help advance the understanding of how racial marginalization embedded in institutions impact youth's well‐being and behavior (Causadias & Umaña‐Taylor, 2018; García Coll et al., 1996; Spencer, 1995). Spencer's (1995) phenomenological variant of ecological system theory further situates youth development, focusing on identity formation (i.e., a developmental task we focus on in this paper) within unique social contexts (Velez & Spencer, 2018). Racially minoritized youth experience marginalizing institutional structures concurrently, impacting youth at multiple levels. The impact of this marginalization (i.e., experiences of racism and other forms of oppression) creates alternate developmental pathways, which we refer to as nondominant pathways or transitions because these experiences are not shared with the White majority (García Coll et al., 1996). These experiences impact racially minoritized youths' development based on their perception of experience or meaning making (Spencer et al., 1997). These self‐perceptions or the creation of meaning within each ecological system influences racially minoritized youths' behaviors, thoughts, and actions (Spencer et al., 1997). Therefore, central to racially minoritized youth's transition to adulthood, and thus the focus of this paper, is their experience within ecological systems. At the microsystem level, we zoom in to examine institutions that most immediately and directly impact development. At the macrosystem level, we zoom out to explore the overarching sociopolitical institutions that influence development. We acknowledge that multiple institutions exist within each ecological system. Still, we limit our inquiry to institutions necessary for transitioning to adulthood: the family, school, and work (at the microlevel), and politics (at the macro level).

We examine policing across each institution and ecological system to emphasize the process of marginalization for racially minoritized youth and to understand their resulting behaviors. Specifically, we focus on how policing and the U.S. institutional systems work together to produce specific youth behaviors in constructing a compliant citizen within a country that privileges White people. These institutions center and normalize whiteness through normalizing a prescribed progression from adolescence to young adulthood which systematically marginalizes racially minoritized youth.^1^ Scholars can misunderstand the normal developmental process of racially minoritized youth by centering and normalizing whiteness. Racially minoritized youth development “requires more explicit attention to the unique ecological circumstances (e.g., the pervasive influence of racism) these children face.” (García Coll et al., 1996, p. 1893). Because these institutions determine and enforce what it means to successfully transition into adulthood through rules, policies, laws, and cultural norms, these institutions may react violently, through policing, when racially minoritized youth deviate from dominant pathways of transitions to adulthood such as school completion, employment, residential independence, partnering, and parenthood (García Coll et al., 1996; Maggs et al., 2012; Settersten, 2007). This dynamic negatively impacts youth's navigation and survival strategies (i.e., reactions) during this transition. We explore policing within each ecological system to (1) identify relevant institutional structures and explore how these institutional structures actively contribute to the marginalization process of racially minoritized youth and (2) juxtapose this racial marginalization against current youth resistance. We acknowledge that this analysis is limited by a focus on racial marginalization even though racially minoritized youth may also be marginalized at multiple intersecting identities (Crenshaw, 2013; Velez & Spencer, 2018).

We posit that a multidirectional and cyclical relationship exists between institutional marginalization and youth resistance. Racial marginalization impacts how institutions respond to youth in transition and how these institutions interpret youth's behaviors and reactions. Youth behaviors and reactions are shaped by the racial marginalization experienced within the respective institutions of family, school/work, and politics. These coexisting structural inequities intentionally disadvantage youth via racial marginalization, creating disparities within each ecological system in the United States (National Academies of Sciences, Engineering, & Medicine, 2017). For racially minoritized youth who also often experience economic and social marginalization, the transition from adolescence to adulthood can be a precarious time. Thus, there is much interest in this transition—interest in developmental opportunities, experiences, and interventions, and these factors' relationship with other educational, socioeconomic, and life outcomes. We emphasize how structural inequality shapes racially minoritized youth behaviors and center the potential for resistance, arguing that a resistance lens allows us to deepen our understanding of the transition to adulthood for racially minoritized youth. To fully understand the lives of racially minoritized youth during this transition period, we need a greater exploration of the contexts these youth live in and the conditions that support or thwart their efforts to resist oppression.

## RELEVANT INSTITUTIONAL STRUCTURES THAT MARGINALIZE RACIALLY MINORITIZED YOUTH

Three main developmental domains critical to transitioning to adulthood include establishing a unique identity and navigating relationships, school to career transitions, and independent living, including financial independence and social mobility. These developmental domains occur across multiple ecological systems, and the power imbalances within their related institutional structures create the need for resistance. These systems are contextual, phenomenological, and person centered (Darling, 2007). Regardless of the impact and application of each system, these systems illuminate how racially minoritized youth must navigate their transition to adulthood through U.S. institutions while experiencing systematic marginalization across those varied institutions. Different parts of the ecological systems apply to each developmental domain. Each domain has dominant institutional expectations and the potential for youth resistance. Dominant institutional responses are often geared toward youth progressing through dominant transitions to adulthood that are disproportionately centered in whiteness. Therefore, the accompanying norms and expectations are based upon the experiences of White youth. These dominant institutional expectations and responses reflect systematic oppression and structural racism to produce “law‐abiding citizens.” Specifically, the purpose of racially minoritized youth's behavior varies as they navigate respective institutional norms (e.g., mandatory school attendance) and the dominant institutional responses (e.g., rewarding perfect attendance, reporting truancy to officials); some behaviors may reflect resistance while others do not. Thus, for racially minoritized youth, resistance may occur at the intersection of being off time (e.g., early or late) in social role transitions and navigating continued marginalization. Resistance comprises youth behaviors that defy the traditional norms or responses encouraged by the systems. From a resistance perspective, racially minoritized youth agency and existence are central (Walsh, 2021), as they respond to the demands and expectations of these systems.

The adapted ecological systems model details how racially minoritized youth's social locations and lived conditions within the different systems from the micro (e.g., relationship, community) to the macro (e.g., sociopolitical) shape racially minoritized youth behaviors. Often, institutions embedded within each system, whether it be the institutions of family, school, work, and politics, may interpret these youth's behavior from a negative normative baseline and thus react in a restrictive or oppressive manner. From an ecological systems perspective, the focus here begins with the individual who moves through transitional experiences associated within the relationship, community, and sociopolitical systems. Within the relationship system, the focus is on the responses of families and friends to youth behaviors, and the community system focuses on the responses of neighborhoods and schools where youth geographically live, learn, and work. Finally, we end with the sociopolitical system, which reflects the broader political economy where youth experience social norms, rules, and expectations on an affective level.

## REFRAMING YOUTH BEHAVIOR AS RESISTANCE TO SYSTEMIC MARGINALIZATION

The transition to adulthood, a critical developmental period marked by the transition from adolescence to early adulthood, often shapes racially minoritized youth's behaviors. Youth who have more economic stability and spare time tend to experience less urgency to become a stable, thriving adult (Sapiro & Ward, 2020). Racially minoritized youth are more likely to be afforded less time, if any time at all, to transition to adulthood (Munson et al., 2013; Sapiro & Ward, 2020). Reduced time to adulthood (Mahadeo, 2019) affects how youth experience and complete unique developmental and self‐actualization tasks critical to this transition. Developmental and self‐actualization tasks for self‐making include exploring one's identity and possibilities, navigating feelings of being in‐between adolescence and adulthood (i.e., not quite being a child or an adult), and experiencing instability (Arnett, 2000, 2006). Youth who have the luxury of elongating the transition to adulthood can focus on achieving these developmental and self‐actualization tasks in different parts of life, such as establishing independent living, attending school, and choosing work opportunities such as internships (Fussell & Furstenberg, 2005).

Some have argued that Arnett's stage‐based formulation of developmental and self‐actualization tasks during this transition to adulthood is flawed theoretically and methodically (Côté, 2014). Our argument here is focused on how stage‐based formulations are not simply neutral descriptives of an age period; formulations such as these are normed for *all* youth in the United States and center whiteness. We suggest that how racially minoritized youth experience the transition to adulthood and engage in behaviors corresponding with nondominant (e.g., accelerated or delayed) transitions to adulthood are key to understanding resistance in this age group within these systems.

### Youth Resistance to Dominant Systems

What constitutes resistance as racially minoritized youth transitioning to adulthood navigate relationships, communities, and politics? Resistance in everyday life is defined as daily actions that are subtle responses to power (Cohen, 2004; Pattillo, 2015; Rosales & Langhout, 2020; Scott, 1985). Under some circumstances, racially minoritized youth's behavior can be subtle resistance or a reaction to power imbalances reflected across systems. Scott (1985) argues that everyday resistance are tactics that exploited people use to survive and undermine repressive domination, especially in contexts where rebellion is too risky. Under other circumstances, racially minoritized youth's behavior can be interpreted as deviance, and deviance can be interpreted as resistance. Cohen (2004, 2010) argues that what many view as deviance in the behavior of Black youth, such as defying dominant norms in their sexual lives and alternative family structures, is resistance. Reinterpreting racially minoritized youth behaviors away from the dominant traditional frameworks of delinquency and deviance to a reflection of resistance emphasizes the agency of youth and their existence within oppressive systems.

Following Cohen's (2004) contention that “not all acts of deviance are examples of politicized resistance” (p.39), but there is “political potential” (p. 39) in everyday acts of deviant behavior that “are not necessarily made with explicitly political motives,” (p. 30), our framework does not argue that all youth resistance is “political resistance.” Instead, we are focused on detailing potential resistance to manifestations of oppression. We explain why these behaviors are observed and the systems' maintenance of these patterns. The current literature largely considers the behaviors and reactions of these youth that challenge authority or reject traditional values as impediments to completing developmental tasks (e.g., engagement in intimate relationships and establishing financial independence). These behaviors can be both impediments to developmental tasks while also being potential acts of resistance. Racially minoritized youth may choose to pursue those more rebellious behaviors *as* acts of resistance to the institutions.

Because racially minoritized youth are embedded in multiple systems, we should expect different behaviors and reactions to dominant institutional norms that are likely embedded in each system. These ecological systems and their corresponding institutional structures and dominant responses to racially minoritized youth are explored and problematized by bringing resistance theories into the analysis. A resistance framework captures the politics of subordinate or marginalized groups. Racially minoritized youth resistance can range from expressing emotions through aggression, to open refusal to cooperate with requests of parents or teachers, to missed appointments, and running away. Racially minoritized youth transitioning to adulthood are among the most precarious, the most vulnerable, and the most overlooked by the state as they disproportionately have marginalizing experiences. For example, racially minoritized youth disproportionately make up the youth who live in poverty (Marks et al., 2020) and are in contact with the juvenile justice system (Curtis, 2013); thus, they are likely to demonstrate their resistance in forms akin to other powerless groups.

Youth may acquire various behaviors in reaction to institutional structures at the relationship, community, and sociopolitical levels. Considering youth as embedded across these prominent systems and the systems' dominant responses, we see some racially minoritized youth behaviors as resistance‐informed reactions based on viewing racially minoritized youth through a resistance lens; these youth are reacting to norms and expectations perpetrated by institutions that privilege whiteness and promulgate anti‐Blackness. Under some conditions, behaviors fall into the category of resistance. Everyday resistance is an integral part of the activities that relatively powerless groups can employ. These acts include “foot‐dragging, dissimulation, desertion, false compliance, pilfering, feigned ignorance, slander, arson, sabotage, and so on” (Scott, 1985, p. 29). Although this excerpt is describing acts within peasant resistance and all these behaviors do not specifically translate to youth, the theoretical link between these types of acts and racially minoritized youth resistance is that they are behaviors that relatively powerless groups can employ by being more subtle and easily deniable.

Thus, resistance is a complicated and broad umbrella concept as we consider different mechanisms, actors, techniques, dynamics, and historical and political contexts (Baaz et al., 2017). Regardless of type, resistance happens according to its proximity to power (Lilja & Vinthagen, 2018). Because of their sociopolitical and economic location and vulnerability, racially minoritized youth in transition, especially those with nondominant transitions to adulthood may not be as likely to engage in visible resistance such as protests, demonstrations, or confrontational expressions that may compromise their safety, stability, and access to necessary resources.

As Hollander and Einwohner (2004) note, there is scholarly disagreement on what can constitute resistance: Does it need to be recognized by those in power, does it need to be coordinated and intentional, and do the people engaging in the act need to see it as a form of resistance? We acknowledge that neither articulating recognition nor intention of resistance is within the scope of our argument. Consequently, considering the ways youth continue to participate and contribute to mainstream society in the face of oppression offers a theoretical framework to emphasize agency and rearticulates the *potential r*egistry of actions for vulnerable populations as these.

In our framework, racially minoritized youth’s acts of resistance are illuminated if we contextualize their behaviors within prevailing stereotypes of institutions. As racially minoritized youth navigate relationships, we may see youth engage in faking their true feelings to avoid conflict, or false compliance when they engage in avoidance of tasks masked by superficial cooperation. One example of superficial cooperation is girls who are encouraged to “just keep it inside” (Way et al., 2018, p. 7) during relationship conflicts. This may further materialize in “someone who doesn't say what she “really” thinks and feels, who is not “too loud” or “too honest” (Way et al., 2018 p. 8).

Furthermore, using 25 years of research with boys of color focused on the processes of accommodation and resistance to patriarchal, heteronormative, and White supremacist ideologies, Rogers and Way (2018) provide interview excerpts from youth that illustrate the abovementioned dynamic; for example, defining themselves as the “hood guy” or embracing societal male stereotypes of toughness demonstrates accommodation (p.321). On the contrary, examples of simultaneous accommodation and resistance can be seen in excerpts such as “African‐Americans [are] like the lowest percentage at graduation. They are only 48% at graduation… I'm not like the average African‐American” (p. 322) and “Even though there's like a lot of stereotypes … either they're like drug dealers or gang bangers… No, since I'm Black I feel like I gotta, you know, achieve something other than that, you know? I've got goals to do” (p.323). As racially minoritized youth navigate school and work, they are cognizant of stereotypes. As such, we can see resistance when youth skip class (the abandonment of duties) or complete tasks slowly at work (foot‐dragging) as well as when youth attend school and desire to be dedicated to work.

Scholars have shown that racially minoritized parents discuss and prepare youth for discrimination, stereotypes, and racial oppression via “the talk” (Anderson et al., 2021). “The talk” emphasizes the way parents provide guidance and tools for minoritized youth to navigate the transition to adulthood, yet this preparation is rooted in resistance as survival. Particularly, Das et al. (2022) demonstrate that Black mothers in their study were more likely to communicate resistance for survival strategies to their sons, which are individual‐focused and short‐term responses to oppression that are sometimes necessary to survive but can also reinforce existing hierarchies as these strategies do not challenge the status quo. Although studies have shown mixed results for the relationship between preparation for bias and youth outcomes, resistance for survival preparation is associated with more negative outcomes such as depression and lower academic grades (Das et al., 2022; Rogers & Way, 2018). For example, Robinson and Ward (1991) refer to dropping out of school as a resistance for survival strategy. Our framework emphasizes that there are different resistance strategies with varying outcomes. Some youth resistance is for survival while other resistance is to directly confront or change existing systems.

As racially minoritized youth navigate politics, they may engage in voting, protests, or other forms of activism. Cohen (2010) documented a historic voter turnout for the 2008 presidential election among Black youth. Latinx and Asian youth also experienced significant increases in turnout between 2004 and 2008 (White youth did not increase their turnout rates during this time). Racially minoritized youth vote but engage in activities beyond voting, especially amidst increasing racial violence and overlapping pandemics. In a 2020 study of racially minoritized youth's views on the recent protests over racism and policing, voting in local elections was the strategy to make racial progress chosen most often by Asian (18%) young adults compared with Latinx (14%), Black (13%), and White (11%) young adults (GenForward, 2020a). Notably, almost a quarter of Black young adults reported participating in recent and ongoing protests and demonstrations in cities across the country; moreover, most young adults across races and ethnicities said various 2020 protests were strongly or somewhat justified. This indicates that voting, protests, and other forms of activism are considered valid avenues of resistance by youth.

Connectedly, in Anyiwo et al.’s (2020) review of current literature on racial and political resistance of racially minoritized youth, they highlight that youth political actions occur across multiple domains such as joining political parties and campaigns focused on racial injustice, using social media hashtags (e.g., #BlackLivesMatter, #IfTheyGunnedMeDown), and engaging in die‐ins to protest police brutality (see Anyiwo et al., 2018; Robinson & Ward, 1991 for further examples of explicitly political resistance). The elaboration of varied acts of resistance at different levels demonstrates that the type of resistance employed is shaped by racial marginalization rooted in U.S. institutions that uphold White supremacist ideologies. Youth defy the traditional norms or responses encouraged by institutions through these individual or collective acts of resistance.

## ECOLOGICAL SYSTEMS AND RACIALLY MINORITIZED YOUTH'S RESISTANCE TO MARGINALIZATION DURING THE TRANSITION TO ADULTHOOD

During the transition to adulthood, racially minoritized youth enter different and new contexts across various institutions that include interactions within the familial, school, and work relationships (Lee & Waithaka, 2017). Traditionally, scholars have measured these domains pertaining to development across five events: establishing an independent household away from family, finishing school, working full‐time, marrying or partnering, and parenting (Benson & Furstenberg, 2006). Yet, scholars have begun to explore the lengthening transition to adulthood over the past several decades and the challenges the new schedule poses for young people, families, and society (see Settersten & Ray, 2010 for an extensive review). Additionally, there has been a shift in contemporary youth's description of adulthood focusing on qualities such as accepting responsibility for oneself, making independent decisions, and becoming financially independent (Arnett, 1998, 2006). What racially minoritized youth experience as they transition to adulthood is considered critical to understanding how social structures are succeeding or failing to support them, especially considering experiences across developmental domains.

Although Lee (2014) describes the transition to adulthood as adulthood deferred, which allows time for extended transition, free from obligations typical in adulthood such as independent living and self‐sustainment (e.g., paying for basic needs by working), racially minoritized youth, particularly those who experience socioeconomic disadvantage, often disproportionately experience accelerated adulthood also known as adultification (Burton, 2007). Accelerated adulthood does not allow for this deferment but instead adultifies youth by premature exposure to adult knowledge and early adoption of adult roles (Burton, 2007). Overall, accelerated adulthood is fraught with more challenges and future disadvantages than those experienced by youth in transition (Lee, 2014) and is more likely to occur within the context of economic hardship (Burton, 2007). Youth can experience earlier exposure to adult knowledge, roles, and responsibilities within the home environment as accelerated adulthood, and racially minoritized youth are also more likely to experience being perceived as adults within larger institutions such as schools and the law through adultification (Gilmore & Bettis, 2021).

Accelerated adulthood and adultification may harm racially minoritized youth's current, potential, and future opportunities by affecting the responsibilities and demands associated with their transition to adulthood. Importantly, accelerated adulthood shapes the kinds of behaviors that are seen. These differential exposures to accelerated adulthood mean that racially minoritized youth often do not experience the same stage‐based transition to adulthood as their White counterparts, and thus institutions influence their experiences in restricted and oppressive ways through their perceptions of these youth as adults. Bowleg (2021) warns scholars that viewing Black people through a deficit lens is based on the use of inadequate tools centered in whiteness. We propose that instead of expecting racially minoritized youth to adopt and mirror *all* the transitional behaviors or roles of White youth, we should consider how the traditional markers for transition such as family, parenthood, or work are centered in whiteness. Thus, we center our reflections on how nondominant transitions such as accelerated adulthood shapes minoritized youth's behavior. According to Spencer (2011), development is bidirectional as individuals respond and engage with their environment. This means we should *expect* resistance in the face of restriction and oppression instead of compliance or submission. These acts of resistance may include alternative markers of achieving developmental and self‐actualization tasks. Institutions must shift their interpretation of racially minoritized youth's behavior from pathology and dysfunction to a resistance‐informed lens. In doing so, resistance may be seen as an appropriate and productive reaction to oppression that might be expected from minoritized youth living in oppressive environments and nurtured instead of punished.

There are dominant responses of institutions in each domain, given expectations for what is considered “normal” (Settersten & Ray, 2010). Racially minoritized youth are expected to achieve traditional roles and accomplish specific tasks in each of those developmental domains. However, existing systems disproportionately increase the difficulty of this developmental process through surveillance and punitive approaches for racially minoritized youth who do not meet expectations. In many ways, the anti‐Blackness embedded within institutions causes the expectations for minoritized youth to move constantly (Gilmore & Bettis, 2021). On the one hand, minoritized youth are expected to meet developmental tasks centered in whiteness. On the other hand, they are developing in environments that create barriers to meeting those same tasks because of institutional anti‐Blackness.

**FIGURE 1:**
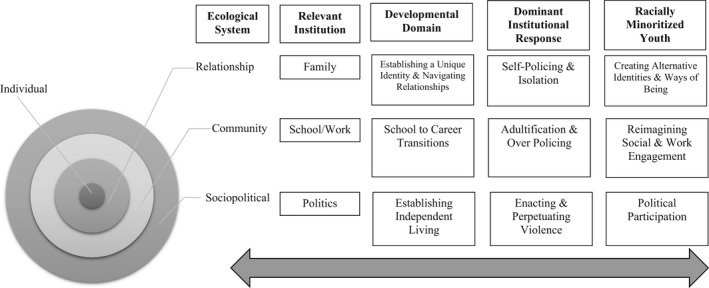
Ecological model of racially minoritized youth resistance during the transition to adulthood.

Minoritized youth and families struggle to navigate institutional anti‐Blackness. They experience overpolicing in school, work, and minoritized communities (Steinmetz et al., 2017) and violence enacted by law enforcement (Cooper & Fullilove, 2016). Even still, parents try to explain these experiences in the broader social context; the racial socialization process where minoritized families help children understand race and racism through implicit and explicit messages. Some scholars argue these messages prepare minoritized youth for bias (Anderson et al., 2021). Others argue this preparation results in minoritized youth overachieving and over complying to manage perceptions and minimize contact with external policing by the state (Anderson et al., 2021; Dow, 2016; Malone Gonzalez, 2019). Although youth react in different ways to these norms of the institutions, attempting to meet developmental and self‐actualization tasks centered in whiteness and engaging in resistance are not mutually exclusive. In fact, there are instances where minoritized youth are attempting to complete developmental tasks *and* may also be showing a level of resistance in everyday ways. Below, we explore three primary developmental domains, dominant institutional responses to racially minoritized youth including potential resultant feelings and perceptions, and then conclude each section with how some racially minoritized youth might behave within each ecological system to recontextualize racially minoritized youth's responses as forms of resistance (Figure 1).

### Establishing a Unique Identity and Navigating Relationships

The first developmental domain as racially minoritized youth transition into adulthood is establishing a unique identity and navigating relationships. Here, the establishment of identity and the relationship system focusing on family, friends, and future partners, are important systems for structuring how the youth experiences and achieves developmental tasks. Additionally, connectedness, or positive relationships with parents, peers, school, community, and other positive adults in these systems is of high value to youth in transition as it promotes adjustment and may buffer against risk factors associated with adversity (Foster et al., 2017). However, the transition from adolescence to young adulthood is filled with rapid changes that could drastically alter those connections depending upon institutional alignment and integrity (Lee, 2014). For those experiencing accelerated adulthood, considering how institutions systematically isolate racially minoritized youth, often characterized by disconnection and misalignment of values, should guide our understanding of their behavior and responses.

#### Self‐policing and isolation

The relationship system can often be characterized by racial socialization in which parents help children understand race and racism through implicit and explicit messages (Anderson et al., 2021) and by a sense of isolation in which youth's relationships may change in new ways including the emergence of increased conflict with parents (Branje, 2018) and romantic interests beginning to form (Connolly & McIsaac, 2009). The process of establishing a unique identity while navigating relationships during racial socialization sometimes focuses on parents teaching racially minoritized youth how to manage perceptions of others. We refer to this behavior as self‐policing and it can result in youth experiencing familial rejection or a sense of isolation. Oppressed populations often police the behaviors, emotions, and appearance of themselves and those within the group to signal to others that they are respectable and safe (Collins, 2009; Harris, 2014). Self‐policing can be out of care or desire to prepare racially minoritized youth for the White supremacist norms and ideologies they will experience such as “the talk” (Anderson et al., 2021). Yet, the consequences of self‐policing or how families police youth emphasize how racially minoritized youth are made aware of the norms and may experience surveillance and punitive measures to encourage these norms within their families.

This self‐policing or families encouraging minoritized youth to manage perceptions by being told to work twice as hard as everyone else to succeed (Mahadeo, 2019), to defer to authority by using “maam” and “sir” (Harding et al., 2017), and to appear less threatening (Dow, 2016) or more acceptable by conforming to professional standards centered in whiteness (e.g., wearing straight hair or talking “proper”; Ferguson & Dougherty, 2022) contributes to racially minoritized youth's identity development (Stevenson et al., 2002). These particular racial socialization messages may or may not be incorporated into racially minoritized youth's identity and future self‐policing. Either way, there are both benefits to aspects of the racial socialization process such as coping, prosociality, and cultural empowerment (Anderson et al., 2018; Stevenson et al., 2002; Wang et al., 2020) and consequences such as chronic stress and resultant health conditions (Doan et al., 2022). Self‐policing may reinforce a sense of isolation from broader society as racially minoritized youth experience the consequences of racial socialization on their time and behaviors that their White counterparts do not.

Given the additional time and effort it takes to adapt to white spaces (Mahadeo, 2019; Yusuf et al., 2022), the family may become a refuge for racially minoritized youth. At home, racially minoritized youth may be able to let down their guard and just be themselves. Family connectedness is one of the strongest protective factors aiding in preventing multiple negative outcomes (e.g., sexual exploitation and violence) for youth (Chisolm‐Straker et al., 2018; Jain et al., 2012; Kessler et al., 2018), yet family connections may change during the transition to adulthood. For some youth, the transition from high school to college, from attending school to work fulltime, or simply turning 18 might mean it is time for them to support themselves financially and/or spend less time with their families. Certain safety nets and basic needs provisions, whether provided by family or the child welfare system, may diminish without the time needed to secure stability as a fully independent adult (Woodgate et al., 2017). These changes in connectedness with their families may contribute to feelings of isolation for racially minoritized youth as there are racial differences in the timing of transitions (Kao & Thompson, 2003), and the success of transitions (Lei & South, 2016).

Marginalization affects youth's identity development as marginalizing systems perpetuates certain identities and traditional roles during transition. As a result, these marginalizing systems can shape racially minoritized youth's self‐perceptions, expectations, and behaviors (Spencer et al., 1997) and their development of sense of purpose (Sumner et al., 2018). Although these processes facilitate the formation of identity, the differentiation and dehumanization as a part of marginalization can limit and maintain how emotions and their respective behaviors are expressed (Lozada et al., 2022). Because racially minoritized youth identity development occurs in the context of racial discrimination, these marginalizing systems can further potentiate racial stress with negative consequences for health and well‐being (Hope et al., 2015). Although marginalization can lead to difficulties with forming a positive and coherent sense of self, greater social isolation, and limited access to mainstream sources of power, it can also help individuals to form close bonds with others through shared experiences and leverage their collective power to work for resistance (Hall et al., 1994).

#### Creating alternative identities and ways of being

Youth's resistance to conditions that produce a sense of isolation and self‐policing may be to create alternative identities and ways of being. Despite growing egalitarian views on gender for racially minoritized youth, especially for girls and women (Lam et al., 2017), familial rejection surrounding racially minoritized youth's gender identity and sexual preference remains a point of contention in families (Richter et al., 2017). In turn, some racially minoritized youth, especially sexual and gender minoritized youth, continue to create new ways of expressing alternative identities and ways of being. They may reject traditional family values and traditionally White familial compositions creating “chosen families” (Hailey et al., 2020) as well as conferring kinship (Scott & Deutsch, 2021). Additionally, some racially minoritized youth create and navigate ambiguous boundaries around supportive networks (Catalpa & McGuire, 2018). Scholars recognize the creation of alternative identities or ways of being (i.e., counter‐narratives) as forms of resistance (Ender, 2019; Wagaman, 2016).

Creating alternative ways of being is seen in resistance as practices that might be replicated by larger organized groups and movements and based on individuals, subcultures, and everyday relations. Although resistance as an act or pattern of actions might undermine or negotiate different power relations, it can also reproduce and strengthen oppressive structures as power holders can mobilize their forces to suppress the resistance (Lilja & Vinthagen, 2018). In one study, Mahadeo (2019) highlights how minoritized youth repositioned themselves to mock systems of oppression (e.g., characterizing Black culture as up to date and White culture as lagging). Thus, creating alternative ways of being for racially marginalized youth has the potential for empowerment through repositioning oneself within society.

### School to Career Transitions

The second developmental domain as youth transition into adulthood is the school to career transition. The following systems impacting youth in transition relates to their community where they attend school and eventually work within. Here, we focus on school to career transitions, given that this is a central aspect of the lives of U.S. youth. Institutional racism and anti‐Blackness make this transition especially difficult for racially marginalized youth. Like any youth, racially minoritized youth are faced with many decisions, such as furthering their education or entering the workforce except they may have to navigate dynamics such as school records with disproportionately high disciplinary marks (Eitle & Eitle, 2004; Morris, 2005) and increased likelihood of discrimination as they seek employment (Pager, 2003). Transitional decisions require the guidance of supportive adults, but also the resources of those adults to shape these trajectories as they make decisions on these pathways to independence that impact their transition to adulthood (Martel, 2021). Additionally, some lack a sense of belonging on college campuses (Hussain & Jones, 2021; Jackson et al., 2022) or in the workplace (Pitcan et al., 2018) due to discrimination. Some racially minoritized youth may feel especially lost or overwhelmed by their current situation or impending future situation at 18 (i.e., age they are no longer considered a minor in most states) due to the tension between disparate opportunities or available options and desires resulting from institutional racism experienced as they completed their primary education and the impending ways they must navigate race and racism.

Youth who may be first‐generation college students, who are disproportionately minoritized youth (Jenkins et al., 2013), may face not knowing how to navigate these new systems. Without guidance from others with college experience amidst navigating predominantly White spaces that may be riddled with microaggressions (Ogunyemi et al., 2020), racially minoritized youth may repeatedly encounter uncertainty, frustration, or a sense of disconnection (Hussain & Jones, 2021; Jackson et al., 2022). Others may not see a way out of their neighborhood and feel trapped to stay in the familiar environment they grew up in despite their desire for upward mobility. Perceived workplace professionalism may shape how racialized minorities behave in the workplace to avoid stereotypes and critique that can hinder promotion (Goodridge, 2022; McCluney et al., 2021). These school to work transitions within U.S. communities are fraught with challenges and barriers as racially minoritized youth navigate being marginalized within schools and the larger society.

#### Adultification and overpolicing

Racially minoritized youth disproportionately experience an acceleration into adulthood due to institutions at the relationship, community, and sociopolitical systems adultifying them. The adultification of racially minoritized youth may impact the guidance offered by adults and shape youth's perceptions of and connections to adults and society at large. In a California study with youth in or aging out of foster care, African American youth reported receiving lower emotional support and advice than their White counterparts (Courtney et al., 2016). Lower emotional support reported is most likely connected to both society's perceptions that Black girls need less nurturing, protection, and support than White girls (Epstein et al., 2017) and that Black boys are seen to be older and more responsible for their actions (and punished more harshly for them) than White boys (Goff et al., 2014). The combined reduced support and lack of acknowledgment of innocence is inherent in the adultification of Black youth and further amplifies the sense of isolation previously described, pushing racially minoritized youth to the margins of society and limiting their time enjoying important developmental experiences of childhood. Even more so, adultification means that institutions prematurely treat minoritized children as adults. This dynamic not only occurs at the relationship system, but also occurs in the community, with schools being a critical site.

The school‐to‐prison pipeline has been well documented for racially minoritized youth (Curtis, 2013; Wald & Losen, 2003). Upon entry into the school system at kindergarten, racially minoritized youth have experienced the overpolicing of their attendance, conduct, appearance, and belongings experienced as suspensions, expulsions, and arrests (Heitzeg, 2014; Henderson & Wyatt Bourgeois, 2021). In fact, many schools employ police, truant, and resource officers and utilize property searches and metal detectors, all as forms of overpolicing (Curtis, 2013). We know this does not happen in all schools but instead disproportionately happens in schools that are racially minoritized (Heitzeg, 2014); many racially minoritized schools are already forcibly suffering from low resources. These marginalizing experiences include the dynamics of adultification and overpolicing and disrupt minoritized children's connection to their school and community, a known developmental asset and protective factor against depression and anxiety for youth (Foster et al., 2017).

If traditional markers of transition are centered in whiteness such as separation from family and independence, school and schooling outcomes becomes a place where racially minoritized youth are pushed to adhere to those norms. In 2017, over half of noninstitutionalized youth who did not enter the military pursued their high school education; those not in school were working (Martel, 2021). Even still, racial disparities exist in high school graduation rates (Quintana & Mahgoub, 2016). Graduating from high school, an outcome often connected to other social determinants of health, is one of the strongest predictors of a higher life expectancy (Olshansky et al., 2012) and lower likelihood of future homelessness (Morton et al., 2018). Additionally, youth are navigating a time when more than a high school diploma or GED is needed to be competitive for entry‐level jobs and starting pay exceeding the minimum wage. As racially minoritized youth transition to adulthood, they are met with barriers to making a living wage to support themselves (and potentially their children and families), alternative and sometimes illegal forms of survival become compelling.^2^ Consequently, schools shape racially minoritized youth's mental, emotional, and material success.

Yet, resistance to these oppressive systems and structures of violence is to be expected. Whether racially minoritized youth submit to or reject these structures, contact with law enforcement in schools or communities is likely due to racial profiling (Vera Sanchez & Adams, 2011). The overpolicing of racially minoritized youth has an enduring impact. Crenshaw et al.'s (2015) report summarizes how Black girls in the United States are “pushed out, overpoliced, and underprotected,” while the Georgetown Law report links the sexual abuse of racially minoritized girls (evidence of underprotection) to their disproportionate contact with the juvenile justice system (Saar et al., 2015). The work in these pivotal reports is furthered by Gadson and Lewis' (2021) exploration of taxonomies of Black women that further help us understand the constant onslaught of gendered microaggressions experienced by Black girls and women. We have recently witnessed these gendered microaggressions play out as outright police violence against Black girls for minor infractions such as “having too much attitude, chewing gum too loudly, and talking “unladylike,’’” (Hines & Wilmot, 2018, p. 63).

Racially minoritized boys are not exempt from this dominant institutional response and in fact are disproportionately overpoliced and incarcerated (Wald & Losen, 2003). The attempts to control racially minoritized boys have extended well beyond the school to prison pipeline and police brutality to civilians in the community. As seen with cases such as the murders of Trayvon Martin and Ahmaud Arbery, examples of adultification and overpolicing were both offered by the defendants and defense to somehow justify their murderous attempts to exert power and control. Being pushed out and overpoliced ultimately results in multiple roadblocks for racially minoritized youth in transition to adulthood as they navigate their schools, communities, and society. The juvenile justice system further exacerbates the trauma experienced by racially minoritized youth. Once in contact with the juvenile justice system, career opportunities and options can become limited because of continued discrimination creating a revolving door of criminalization and marginalization. Illustratively, Currie et al. (2015) highlight this by noting “[w]hile there may be some short‐term “effects” from our massive investment in incarceration, the best evidence suggests that it has deepened the roots of violence and injustice by making poor communities poorer and an unequal society even more so.” (p. 6). Together, overpolicing hinders the developmental self‐work necessary during this transition (Adams, 2021; Vaughans & Harris, 2016). Once criminal involvement has been documented, racially minoritized youth may be further marginalized from their communities and society as adults by being pushed out of housing and the workforce, further perpetuating their contact with the law enforcement system (Evans et al., 2018; Geller & Curtis, 2011; Gowan, 2002; Sugie, 2018).

Lastly, racially minoritized youth interactions with the school system occur during a time when youth are most vulnerable to exploitation. This is particularly important for racially minoritized youth who are disproportionately living in neighborhoods with lower Child Opportunity Indices (i.e., a calculation of neighborhood factors important to child development; Acevedo‐Garcia, Noelke, McArdle, Sofer, Hardy, et al., 2020; Acevedo‐Garcia, Noelke, McArdle, Sofer, Huntington, et al., 2020). In neighborhoods such as these, alternative means of survival can be the norm that contribute to their accelerated adulthood experience (Gwadz et al., 2009; Ogbu, 2013). While school and neighborhood quality vary significantly by race‐class differences, these disparities reflect access to basic needs and ultimately impact life expectancy for racially marginalized communities across the United States (Acevedo‐Garcia, Noelke, McArdle, Sofer, Hardy, et al., 2020; Acevedo‐Garcia, Noelke, McArdle, Sofer, Huntington, et al., 2020). The school‐to‐prison pipeline and the accompanying effects for employment opportunities, lived conditions, and exposure to exploitation contribute to the likelihood of sociopolitical institutions adultifying and overpolicing racially minoritized youth's behavior. Racially minoritized youth are forced to reimagine how to socialize and safely make a living to survive, that is, to imagine just futures where roles and norms center their racially minoritized experiences in direct opposition to centering whiteness. The creation of oppositional identities is not only practical, but a form of resistance (Ogbu, 2013). Relatively powerless groups often employ activities such as false compliance or feigned ignorance, and we add self‐care and by extension communal care as forms of resistance. As bell hooks, (1989) stated, “Oppressed people resist.… by defining their reality.” (p. 43).

#### Reimagining social and work engagement

The youth's resistance to these system conditions is to reimagine social and work engagement. This decade has seen a rapid shift in the way we engage socially via social media (i.e., influencers) and generate income (i.e., gig economy). The access to fame and fortune, described as microcelebrity (Senft, 2013), has changed the way youth approach both work and social engagement. Youth in transition demonstrate resistance by challenging the very way people socialize, work, and learn by engaging in entrepreneurship and by leveraging virtual spaces and technology.

Resistance can be protest or voting or more subtle acts like those we see in everyday life. It is important to note that those under 18 cannot participate in the most traditional form of politics, voting, as they are not eligible which means that even politically racially minoritized youth must find alternative ways to engage. Despite this, there are several ways that they can be civically engaged. They can engage online by amplifying their voice through social media (Wilf & Wray‐Lake, 2021) and in person through volunteering with civic and political organizations, being involved in their school communities, consuming political information, and having political discussions (Holbein et al., 2020). Even as they are eligible and enter the 18–24 age range, these youth are disproportionately burdened with registration laws as they are more likely to change their address or interact with government agencies (Grumbach & Hill, 2019). Scholars have long documented that the time young citizens spend in school is strongly related to their levels of civic engagement later in life (Campbell, 2006; Verba et al., 1995). Because of the lived conditions for racially minoritized youth in transition, the school is a less consistent site for developing these habits.

Civic engagement may not seem immediately relevant to resistance in school or work; however, it is the places where civic engagement falls short or fails to address disparate power imbalances that creates the potential for resistance in everyday life. Practical resistance to oppression can include low‐profile techniques that happen in their ordinary life, such as silence or complaining. In their review of sociological research that engages the concept of resistance, Hollander and Einwohner (2004) outline a range of actions and contexts where resistance can exist, whether it be in the workplace through “bitching” (Sotirin & Gottfried, 1997), changes in appearance through women changing hairstyles (Weitz, 2001), or denying the norms in the family unit (Moghissi, 1999).

### Establishing Independent Living

Like the other developmental domains, the final developmental domain as youth transition into adulthood is independent living, where establishing financial stability and social mobility are essential to achieving this developmental task is centered in whiteness. Oftentimes, racially minoritized youth resist excessive individualism (Robinson & Ward, 1991). The likelihood racially minoritized youth can start living independently and completing the transition to adulthood is dependent on their experiences in other domains. Establishing independent living is not separate from the other developmental domains and the institutional responses from the respective systems, but an accumulation of the advantages and disadvantages perpetuated through the different systems.

Social mobility for youth from low socioeconomic backgrounds is fraught with multiple challenges (Chen et al., 2015). Racially minoritized youth living in economically disadvantaged neighborhoods may experience more maltreatment (Coulton et al., 2007) or reduced opportunities (Acevedo‐Garcia, Noelke, McArdle, Sofer, Hardy, et al., 2020; Acevedo‐Garcia, Noelke, McArdle, Sofer, Huntington, et al., 2020), complicating their trajectory to becoming independent adults. This cumulative effect is most visible in, but not unique to, the sociopolitical system. Especially at the sociopolitical level of the ecological systems model, independent living, financial security, and social mobility are primary developmental aspirations for youth in transition. Racially minoritized youth disproportionately experience unforeseen challenges such as inability to afford the requirements to get an apartment (e.g., security deposit or first month's rest), lack of access to credit, banking, and savings or limited friend or family networks to assist with the transition to moving out or handling unexpected emergencies (GenForward, 2020b). Youth in transition who successfully establish independent lives unfortunately continue to experience the structural violence embedded in U.S. policies. Research demonstrates race (among other identities) influence how societal institutions treat racially minoritized youth through the context of housing via residential segregation (Massey & Denton, 2019), financial vulnerability via persistent wealth inequality (Oliver & Shapiro, 2013), and interpersonal discrimination via microaggressions (Hope et al., 2015). Racial discrimination can shape how racially minoritized youth transition to adulthood (Spencer, 1995). On the one hand, racially minoritized youth are socialized to believe that if they work hard enough, they will achieve financial stability, social mobility, and independence; this is the work of White supremacy, normalizing whiteness. On the other, the harsh reality that they are not exempt from racism, discrimination, and violence can be disheartening.

The marginalization and oppression experienced by racially minoritized youth is embedded in the larger political economy of society. King and Smith (2005) demonstrate that scholars studying the U.S. political economy should center racial inequities as they have been and remain features of the U.S. experience. As a result, racially minoritized youths' political economy also looks different from their White contemporaries. Illustratively, Black and Latinx youths' financial lives differ from their White and Asian American peers. Black and Latinx youth have fewer liquid savings, on average, which leaves them with a smaller financial cushion to turn to in an emergency or an unexpected event (GenForward, 2020b). They are more likely than White and Asian American youth to say they have unmanageable and high‐cost debt, which hinders their ability to access low‐cost debt in the future and build wealth through other means (GenForward, 2020b). These disparities in financial health outcomes result from a long history of systemic racism and discrimination. Other stressors that tend to couple with economic disadvantage exacerbate these existing financial disparities.

#### Enacting and perpetuating violence

The overarching institutions within the sociopolitical system may vary by cultural contexts as geographic location, socioeconomic status, and other characteristics may differ. However, racially minoritized youth often experience similar political contexts. Oftentimes, policy can be characterized by violence. At the turn of the century from 2005 to 2012, Black boys and men were killed by police officers at three times the rate of their White counterparts and were twice as likely to be unarmed when killed (Johnson et al., 2014). Black girls and women are not exempt from police brutality as we have recently seen in the tragic deaths of Sandra Bland, Rekia Boyd, and Brianna Taylor as well as the sexual and physical violence enacted against countless and often nameless girls and women (Lawson, 2018). This violence can be seen within the different systems, from violence within our own households and communities (i.e., domestic violence and self‐policing) to our sociopolitical institutions such as school, the workplace, and politics. The individual disparities described above are in fact a reflection of the broader sociopolitical system. The sociopolitical system is influenced by a dynamic interplay of interactions colliding on all systems. Therefore, we must consider political action as a force of resistance to this marginalization and oppression.

Historically, despite contextual and cumulative disadvantages, marginalized people still engage in resistance against sociopolitical systems. For example, Francis (2018) notes that “racial violence does not suddenly occur and strike at certain moments – it is a foundational component of American politics. Despite noteworthy civil rights victories, black bodies have never been safe from private and state terror in the United States” (p.134). Racially minoritized youth in transition, akin to other marginalized people, engage in resistance to the political institutions at this level such as by advocating for change in laws and policies surrounding policing and the legal system. An uncontested finding is that individuals with more resources, time, skills, and money are more likely to participate in politics (Verba et al., 1995); however, limited resources does not preclude participation. It underscores that barriers to participation may be higher for marginalized people. There are historical and continued efforts to disenfranchise voters of color through restrictive voting laws and voter suppression (Anderson, 2018) that characterize political institutions at this level. Ultimately, an examination of the sociopolitical level emphasizes that we should be concerned about how context and power determine the conditions in which we see actions occur and the type of actions youth take to resist oppression.

#### Political participation

Racially minoritized youth's resistance to these system conditions is to engage in political participation such as organizing in their community, protesting, or even voting if they are eligible. Unlike resistance at the other levels, these acts of resistance are explicitly political, collective, and intentional. The long‐standing history of structural racism and racial capitalism, the Great Recession, and now the COVID‐19 pandemic, threatens youth's lives and places disparate burdens on racially minoritized youth, thereby exacerbating existing burdens of systems and contributing to their persistence. Yet, despite this, more than half of Black (56%) and Latinx (51%) youth are optimistic about their financial futures (GenForward, 2020b). Scholars argue that “part of this optimism is centered on the belief that individuals can better their condition” (p. 28). Consequently, if youth believe that individuals can better their futures, it behooves us to consider the different registries for youth behaviors. Not all registries of action are equally visible, but all are important in communicating their response to systematic marginalization.

A resistance framework offers a way to contextualize and reexamine conflict regarding how social structures, the State, cultural values, and historical practices help shape the political action types. In response to the countless people—Mr. George Floyd, Ms. Breonna Taylor, Mr. Freddie Gray, and others—who have died at the hands of a system meant to serve and protect society, some oppressed groups employ violent acts of political action such as riots, rebellions, and revolutionary movements instead of less violent forms such as petitions, rallies, and boycotts. Racially minoritized youth, particularly Black youth, are increasingly viewing revolution as a viable solution to make racial progress (GenForward, 2020a). Although respondents do not describe the kinds of actions required for a revolution, violent acts of political action and traditional ones may be seen as valid responses to oppressive systems. Acts of violence are understandable as means of resistance for oppressed groups as these groups often have limited access to mainstream sources because of marginalization.

Scholars have found that protest activity, whether mass movements or individual actors, can contribute to movement building, especially for underrepresented groups. For example, Williamson et al. (2018) found that Black Lives Matter protests are more likely to occur in localities where police have previously killed more Black people (Williamson et al., 2018). Relatedly, Towler et al. (2020) demonstrated that Colin Kaepernick's protest of police violence and activism mobilized Black Americans to political action. These findings show that individuals do not live in a vacuum, with protests having spillover effects in other political lives. Gillion and Posey (2019) demonstrate that minority protests have electoral outcomes that influence who runs and gets elected.

### Summary across Developmental Domains

Ultimately, we can summarize resistance across the developmental domains in three ways. First, youth are resourceful—they create alternative identities and ways of being through their creation and participation in subcultures that defy the dominant institutional expectations. Second, youth imagine just futures—restrictions placed upon racially marginalized youth transitioning between school and workplace opportunities creates the need to imagine roles and norms that center on their racially minoritized experiences instead of centering on whiteness. They choose how they engage socially and with the workforce, giving them a sense of empowerment and agency to control their lives and how they spend their time next. Youth behavior can reflect this form of resistance through being opinionated or outspoken about the status quo, resulting in these youth reimagining their social and work engagement. Finally, racially minoritized youth engage in political participation. Scholars often focus on the system conditions that may contribute to behaviors reflecting youth accommodating or internalizing dominant ideologies. Our work emphasizes that there is the potential for resistance; youth can choose to exit political, social, and economic society, and some choose to maintain engagement despite the disproportionate burdens of their life. A resistance lens offers a way to deepen our understanding of the complex transition to adulthood for racially minoritized youth.

## RESISTANCE‐INFORMED RESPONSES—REFLECTIONS ON CLINICAL AND RESEARCH IMPLICATIONS

A resistance framework provides one way to see the experiences of racially minoritized youth transitioning to adulthood, especially those experiencing nondominant pathways of transition such as accelerated adulthood and the areas where they resist oppression and the status quo. Below, we present lessons from community‐based youth justice programs identified by Myers and Goddard (2013) that reflect using an alternative lens of resistance. They identified four salient threads found in the model programs they analyzed: (1) help youth navigate the realities of their neighborhoods, including mistreatment by law enforcement and race‐based social and economic inequalities; (2) disrupt the overcriminalization of young people of color; (3) educate youth by explicitly connecting structural violence to the perpetuation of poverty; and (4) increase youth's understanding of the origins of social and economic inequality by connecting this knowledge to their unique experiences (Myers & Goddard, 2013). This analysis provides meaningful lessons when considering the kinds of resistance racially minoritized youth in transition use. Collectively, these lessons underscore areas within each ecological system in which we recommend viewing racially minoritized youths' behaviors as resistance.

### Rethinking Risk and Safe/Brave Spaces for Youth‐Led Self‐Making

First, as practitioners and researchers recognize racially minoritized youth's ability to be resourceful, emphasizing youth access to the information they need to impact change in their lives becomes a priority. Given the resourcefulness of youth in transition, we recommend rethinking risk by shifting our focus to the socioeconomic factors that entrap youth instead of labeling them with deficit‐based terms (Bounds et al., 2020; Marks et al., 2020). Goddard and Myers (2017) further argue that shifting from oppressive, risk‐based assessments, and interventions is sorely needed. This expectation of compliance and the practices of over‐pathologizing and criminalizing can make practitioners, researchers, and other scholars complicit in racially minoritized youth's oppression.

Given the challenges described above with the advancement of social media, leveraging healthy digital spaces for youth that amplifies healthy versions of themselves is sorely needed. Music, in particular rap, has a long history of the use of meaning‐making and resistance for oppressed people (Anyiwo et al., 2022; Martinez, 1997). Adams (2021) argues that racially marginalized youth need a safe space for self‐making:

Youth being youth will test boundaries, explore, be spontaneous and run afoul of authority. Black youth struggle with a paucity of safe spaces to do this work, and the relative unavailability of healthy role models to emulate in the work of self‐understanding. Such spaces offer opportunities to take ethically informed developmentally appropriate risks without dire consequences. (p.60)

Stornaiuolo and Thomas (2018) cite restorying as a political action that facilitates self‐making and world‐making, thereby creating counter‐narratives for racially minoritized youth. The process of restorying allows youth to “reshape perspectives and experiences that have been routinely marginalized or silenced (Stornaiuolo & Thomas, 2017; Thomas & Stornaiuolo, 2016)” (p. 346). These counter‐narratives allow for the recontextualization of racially minoritized youths' lives giving voice to contexts and perspectives that have been omitted by systematic marginalization (Stornaiuolo & Thomas, 2018). The facilitation of critical consciousness and feelings of empowerment are crucial to developing positive identities and the self‐efficacy of racially minoritized youth (Hipolito‐Delgado & Zion, 2017). Building upon the strengths of racially minoritized youth through amplifying their voices through meaning making in safe spaces and/or brave spaces facilitates identity and relationship development for youth who may not have had the full opportunity to develop in this developmental domain. While safe spaces and brave spaces are both needed, they differ. Safe spaces focus on racially minoritized youth's safety where they might be free from marginalization and violence, often requiring hypervigilance and high levels of emotional self‐regulation (Adams, 2021). In brave spaces, however, racially marginalized youth can respectfully engage, dialogue, and interact with controversial issues where ownership and acceptance of intentions, choices, and the impact of those intentions and choices can be challenged (Ali, 2017). With youth's increasing engagement online, social media has become a primary site for all youth to create counter‐narratives through restorying, build community, and develop critical consciousness and collective action (Wilf & Wray‐Lake, 2021).

### Youth‐Adult Partnerships & Scaffolding Transitions

Second, some youth transitioning into adulthood, particularly those who have experienced maltreatment, may have a stronger desire to exert their independence from their families or other figures of authority—a developmentally appropriate sense of empowerment and agency to control their lives and how they spend their time next (Godoy et al., 2020; Sahl & Knoepke, 2018; Samuels & Pryce, 2008). Youth behavior can reflect this form of resistance through being opinionated or outspoken about the status quo. However, we must proceed with caution as the adultification of Black children has reinforced the fact that these youth often grow up way too soon. Studying the impact of adultification further to understand our youth's needs during this transitional period is crucial (Epstein et al., 2017). Bounds et al. (2020) offer specific recommendations for working with youth with socially complex needs that emphasize youth independence while balancing their need to belong (Lee & Berrick, 2014). They, along with others, argue for youth–adult partnerships (Bounds et al., 2020; Godoy et al., 2020; Sahl & Knoepke, 2018). In youth–adult partnerships youth engage in higher levels of participation as collaborators in decision‐making and governance (Ramey et al., 2017). Adults can help youth develop desired identities through this self‐reflection and acknowledgment of their unique experiences. For example, if the goal is to cultivate a positive American identity, Spencer (2011) states:

Rather than perpetuating this distorted picture of vulnerability and privilege, our goal should be to help young people to understand who they are, what their resources are, how historical forces have contributed to their lives, and what they can offer society as agents of change, promoters of social justice, and salient sources of diverse strengths. (p. 10)

These youth–adult partnerships can highlight the social truths youth experience and disrupt how youth see themselves within marginalizing systems. Racially minoritized youth lose time to physical, psychic, and emotional labor required to process racialization and racism (Mahadeo, 2019) as a result intentional scaffolding is specifically needed for racially minoritized youth to explore the different pathways they experience while authentically confronting marginalization, grounding their experiences within historical context, and reinterpreting their unique contributions to society as valuable. The explicit highlight of social truth allows for it to be further analyzed and corrected (Spencer, 2011). In doing so, we may facilitate unique achievements of important developmental and self‐actualization tasks that generate a solid sense of purpose and direction; in addition, we can start to rectify associated inequalities, creating the potential for collective mobilization against the problems (e.g., we may learn something from these partnerships that help foster resistance).

### Cultivating Youth Activism

Finally, youth have different responses to structural inequality perpetuated by institutions. Some racially minoritized youth continue to participate in and contribute to society, while others choose to exit political, social, and economic society. Even still, some may respond in a combination of remaining and exiting. Ultimately, racially minoritized youth's responses to dominant institutional barriers will vary and change as they transition into adulthood. The continued barriers create the potential for resistance to emerge. More research is needed on how resistance can operate as a survival and coping strategy against oppression and further marginalization as well as a resistance for liberation (Robinson & Ward, 1991) that aims to center racially minoritized youth experiences and disrupt the systems of marginalization. Additionally, research is needed on how to best incorporate existing resistance strategies used by racially minoritized youth (and for what purpose) in existing interventions to promote activism and resistance strategies to cope with oppressions. Racially minoritized youth voice should be central to determining when and what strategies to use and for what goals. Facilitating youth activism has the potential to not only encourage youth's continual participation in society, but it may also nurture racially minoritized youth's sense of acceptance, self‐worth, and future participation in the political process as scholars have shown (e.g., Aspholm and Mattaini (2017) cite using the cultivation of youth activism as a pathway to violence prevention and Spencer (2011) highlights cross‐generational analysis as a strategy for positive American identity formation). Youth activism has the potential to enact meaningful change for us all.

## CONCLUSIONS

We demonstrated that some racially minoritized youth, especially those experiencing accelerated adulthood, might exhibit resistance in response to institutions encouraging norms and behaviors that center whiteness. The persistence of these dominant norms and behaviors systematically marginalizes racially minoritized youth as these institutions define what it means to transition into adulthood “successfully.” When racially minoritized youth deviate from the dominant pathways to adulthood, the dominant institutional responses are to react violently through policing. Thus, racially minoritized youth resulting behaviors reflect resistance to these dominant structures. We highlight approaches that consider ways to foster continual participation and engagement at each ecological system level in light of oppression by using a resistance‐informed lens. In doing so, we encourage authentic engagement with youth in self‐making in safe and brave spaces, youth‐adult partnerships and meaningful scaffolding, and cultivating youth activism. Examining dominant institutional structures and racially minoritized youths' reactions to the oppression inherent at each system level offers a new way to evaluate youth's reactions and leverage their strengths in new ways.

## Footnotes

^1^
Arnett (2000) uses the term emerging adulthood to refer to the transitional period between adolescence and young adulthood. Arnett (2000) does not specifically address race as a factor, but instead references this as a phenomenon in developed countries. We apply García Coll's (1996) and Spencer's (1995) ecological framework of minority youth development to emphasize marginalizing experiences across ecological systems (i.e., the integrated model). We use Schooley's et al. (2019) definition of whiteness as “a set of often unnamed and unmarked cultural and racial practices (e.g., customs, traditions), values, and attitudes that signify what is considered dominant, thus privileging White skin and naturalizing systems of White supremacy” (p. 532). Specifying how whiteness is centered and normalized in how transitions to adulthood are understood places an important emphasis on the role of systems of oppression across institutions, systems, and contexts. Our framework affords theoretical space to incorporate varying experiences and perceptions of experiences.

^2^
In 2019, the living wage in the United States was $16.54 per hour, or $68,808 per year, before taxes for a family of four (two working adults and two children); the minimum wage does not provide a living wage for most American families (see Glasmier's, 2021 article on her *Living Wage Calculator*).
